# Hybrid imaging is the future of molecular imaging

**DOI:** 10.2349/biij.3.3.e49

**Published:** 2007-07-01

**Authors:** RJ Hicks, EWF Lau, DS Binns

**Affiliations:** Centre for Molecular Imaging and Translational Medicine, Peter MacCallum Cancer Centre, Victoria, Australia

**Keywords:** FDG, PET, SPECT, tomography, hybrid imaging, oncology

## Abstract

Correlative imaging has long been used in clinical practice and particularly for the interpretation of nuclear medicine studies wherein detailed anatomical information is often lacking. Previously, side-by-side comparison or software co-registration techniques were applied but suffered from technical limitations related to the differing geometries of the imaging equipment, differences in the positioning of patients and displacement of mobile structures between studies. The development of the first hybrid PET and CT device struck a chord with the medical imaging community that is still ringing loudly throughout the world. So successful has been the concept of PET-CT that none of the major medical imaging manufacturers now offers stand-alone PET scanners. Following close behind this success, SPECT-CT devices have recently been adopted by the nuclear medicine community, already compelled by the benefits of hybrid imaging through their experience with PET-CT. Recent reports of adaptation of PET detectors to operate within the strong magnetic field of MRI scanners have generated further enthusiasm. Prototype PET-MRI devices are now in development. The complementary anatomical, functional and molecular information provided by these techniques can now be presented in an intuitive and aesthetically-pleasing format. This has made end-users more comfortable with the results of functional imaging techniques than when the same information is presented independently. Despite the primacy of anatomical imaging for locoregional disease definition, the molecular characterisation available from PET and SPECT offers unique complementary information for cancer evaluation. A new era of cancer imaging, when hybrid imaging will be the primary diagnostic tool, is approaching.

## INTRODUCTION

At medical schools throughout the world, doctors in training are taught the basic sciences of anatomy, biology, physiology, biochemistry, and so forth. However, when it comes to diagnostic imaging, there is a heavy reliance placed on tutelage regarding anatomically-based techniques while molecular and functional techniques are often given scant attention. Most clinicians are therefore very comfortable with using CT, X-rays, ultrasound and MRI because the output is readily understood, particularly if the images are accompanied by judiciously placed arrows demonstrating the abnormalities. Nuclear medicine techniques, apart from bone scanning, which resembles sufficiently the mind’s eye perception of a skeleton to pass as an anatomical representation, have often failed to capture the imagination of the clinician. There is a feeling that functional imaging is somehow less valid than radiological techniques because of the lack of fine spatial resolution. The term “unclear medicine” is a common derogator used by clinicians when discussing the work of nuclear medicine imaging specialists. Perhaps this partly reflects the complexity of the principles that underpin these techniques and that often do not lend themselves to “gestalt” interpretation. Time-activity curves, deconvolutional analysis and compartmental modelling are all based on mathematics and are generally held to be beyond common understanding.

Nevertheless, the general public deals with functional information on a daily basis and can integrate it into decision-making processes if it is presented in an intuitive format. An obvious example appears in weather reports on television or computer screens where serial radar images of rainfall are displayed over a map of a geographical region with appropriate labelling of landmarks. Different colours are used to display the intensity of precipitation and a cinematic function is utilised to demonstrate the movement of a storm system across the land. The end-user can easily decide whether to take an umbrella on their walk, or to bring in their drying clothes from the washing-line on the basis of such information without understanding the complexities of radar, global positioning systems, satellites or meteorology. Hybrid imaging provides a similar intuitive integration of information from functional and structural imaging techniques without requiring a detailed understanding of the technologies needed to produce them.

## THE HISTORY OF HYBRID IMAGING

**Figure 1:**
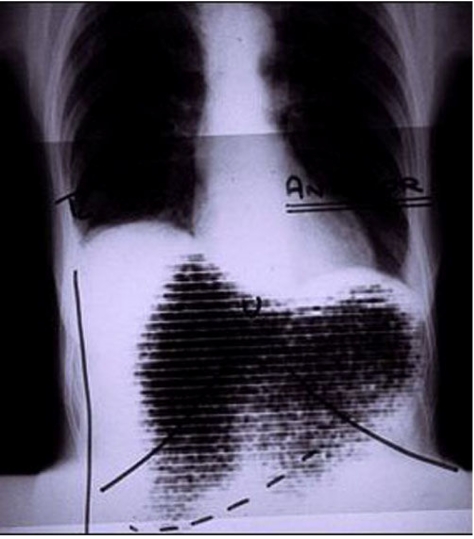
A fusion of a rectilinear scan using I-131 rose Bengal with a chest X-ray outlining the position of the diaphragm was used to diagnose subphrenic abscess before the availability of CT scanning and ultrasound. This represented one of the first examples of “anatamometabolic” imaging.

Nuclear medicine actually started as a non-imaging specialty with probes used to measure radioactivity in restricted regions of the body, such as the thyroid gland. The first practical form of nuclear medicine imaging was that performed on rectilinear scanners [1]. These devices had a very small scintillation detector that moved methodically over the body to produce, usually with a 1:1 scaling, dots on paper or, later, film proportional to the number of radioactive events recorded in a particular unit of time. Because the resulting images were “life-size”, they could be easily overlaid on plain X-rays, which were also acquired without magnification. This fusion of X-rays and rectilinear scans was used, for example, to diagnose subphrenic collections prior to the availability of CT scanning (Figure 1). Clearly, “anatamometabolic” imaging is not a new concept.

The development of the gamma camera by Hal Anger increased the capacity for imaging larger regions of the body simultaneously but limited the ability to perform direct correlations with anatomical images because the film or computer-generated images were usually minified. However, with the development and increasing clinical application of tomographic imaging techniques such as CT and SPECT in the 1980s and 1990s, interest in image fusion increased. Being computer-generated and therefore of an intrinsic digital format, the images from these tomographic techniques were amenable to software manipulations including translation, rotation, rescaling and, potentially, to deformation. Because of the lack of deformation in brain structures, software solutions for image co-registration were most successful for neurological applications [2, 3]. However, even early in the development of SPECT, fusion of anatomical and functional information was performed to assess the distribution of radioligands that provided limited anatomical information in their own right. Examples include radiolabeled monoclonal antibodies [4, 5].

Relatively early in the development of clinical PET in oncology the advantages of correlating structural and metabolic information were also recognised [6]. PET co-registration with both CT and MRI found direct clinical application in the guidance of neurosurgery [7]. It was also found to improve the accuracy of staging lymph node involvement in patients with non-small cell lung cancer (NSCLC) [8] and was used in radiotherapy planning [9].

**Figure 2:**
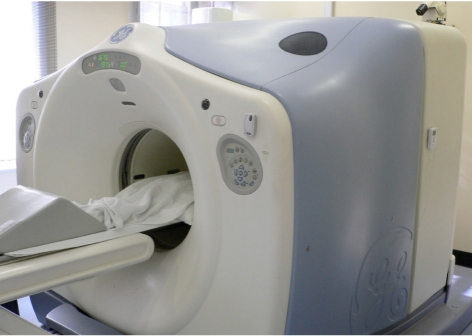
The Discovery LS (GE Medical Systems, Milwaukee, WI) was the first commercial PET-CT scanner to become available. This scanner at these authors’ institution was installed in 2001 and has now performed over 12,000 scans. Implementation of this scanner involved end-by-end installation of a 4-slice Lightspeed plus CT with the GE Advance NXi PET scanner with a new patient bed. The CT and PET components operated on independent computer platform.

From the concept of image registration and fusion, it was a relatively short intellectual leap of faith to develop hybrid scanners. GE Medical Systems first commercially released a SPECT camera with an X-ray source capable of providing low-dose CT images. This device, which went by the trade name “Hawkeye”, immediately found application in oncology [10, 11]. Although this iteration of SPECT-CT was available slightly earlier before PET-CT in a commercial sense, the CT on the Hawkeye was of suboptimal diagnostic quality and added significantly to the total acquisition time of a routine SPECT study. The development of combined PET-CT systems integrating a diagnostic CT by Townsend and co-workers has revolutionised hybrid imaging [12]. Early versions of these hybrid devices incorporated partial-ring PET scanners but further development has led to state-of-the-art PET and multi-detector CT (MDCT) now being integrated into current devices [13]. The first combined PET-CT to be available commercially was the Discovery LS scanner (GE Medical Systems, Milwaukee, WI). This device pragmatically involved simply bolting what were then current generation PET and MDCT devices together and using a newly-designed patient bed (Figure 2).

## RECENT DEVELOPMENTS IN HYBRID IMAGING

Subsequent developments have seen the advent of PET-CT designed as an integrated hardware and software platform. Such has been the success of hybrid PET-CT scanners that none of the major manufacturers currently offers stand-alone PET scanners for commercial sale. Recent advances include incorporating 64-slice MDCT, new detector technologies and development of time-of-flight scanners [14, 15]. The rapid take-up of PET-CT in clinical practice has generated significant economies of scale to be realised in the manufacture of systems and led to a significant fall in the cost of these systems or availability of more technologically-advanced systems at comparable prices. It has also stimulated reinvestment in instrumentation research and development, which had stalled for many years as PET languished as a primarily research tool with a low installed base. Accordingly, significant improvements in performance with respect to resolution and scan acquisition times are expected in the near future.

**Figure 3:**
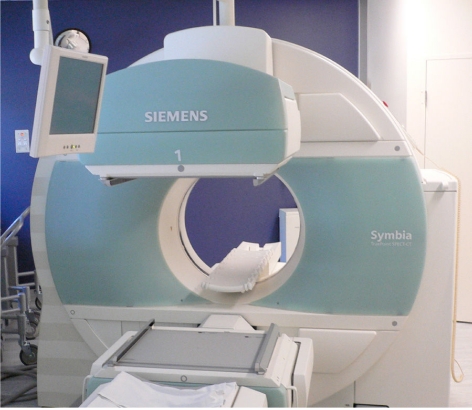
Modern hybrid SPECT-CT scanners incorporate multi-detector CT. This is the Siemens Symbia 6 system installed in these authors’ facility.

Following the success of PET-CT scanners with integrated diagnostic MDCT, Philips and Siemens (Figure 3) both subsequently introduced SPECT-CT scanners that also included a diagnostic MDCT. The superior diagnostic quality of the CT on these scanners, particularly for soft tissues in the abdomen, and the much shorter CT acquisition times offer significant advantages over the GE Hawkeye system. However, these systems are more expensive and involve higher radiation doses [16]. Hence the choice of system is highly dependent on the range of applications for which the scanner will be used.

Despite the success and popularity of PET-CT and, more recently, of SPECT-CT, there are some shortcomings in the use of CT as a complementary anatomical imaging modality. Firstly, CT adds radiation dose to the overall examination, particularly if used in a full diagnostic role [17]. Secondly, CT provides relatively poor soft tissue contrast in the absence of oral and intravenous iodinated contrast, particularly if low-dose acquisition protocols are utilised to minimise incremental radiation exposure. These two theoretical limitations do not apply to MRI, which does not involve ionising radiation and provides soft tissue imaging with high spatial resolution and superior contrast compared to CT. MRI can also provide more advanced ‘functional’ techniques such as diffusion and perfusion imaging as well as spectroscopy, which may be complementary to functional information obtained by PET. Furthermore, the high sensitivity of PET may also complement the poor signal strength inherent in current functional MRI imaging. The combination of PET and MRI into a single scanner may therefore prove to be the ultimate hybrid imaging modality, combining the metabolic and molecular information of PET with the excellent anatomical detail of MRI, while offering new potential applications with respect to functional MRI techniques [18].

There have been several recent publications on the value of fused or co-registered PET-MRI images in pre-clinical and clinical practice (19-22). There are, however, a number of technical problems to be overcome before a hybrid PET-MRI scanner can become a reality. Both MRI and PET have the potential to affect each other’s performance in their current form. One of the main problems is that the photomultiplier tube, a fundamental component of current PET detectors, will not function in a ‘magnet’ as the high magnetic field causes electrons to deviate from their original trajectory, resulting in loss of gain. A small prototype PET-MRI scanner has been developed using long optical fibres to transport light from the detector to photomultiplier tubes situated in a low field region [23].

A small 5.4 cm diameter MRI-compatible PET scanner operating within the bore of a 9.4T MRI spectroscopy system and a 4.7T small bore animal MRI imaging system have been developed successfully. There is however significant light loss in the long optical fibres, leading to poor energy and time resolution. This design is likely to be impractical for a large number of detectors since the large volume of optical fibres required would probably significantly limit the axial field of view. A more promising alternative is the use of avalanche photodiode (APD) technology, which is a compact and reliable silicon-based device. APDs have successfully replaced photomultiplier tubes in a high resolution PET system, and can function in high magnetic fields of up to 9.4T without any performance degradation. A performance test of an APD-based LSO PET detector in a 7 T animal research MRI scanner yielded encouraging results [24]. There was only slight degradation of the PET detector energy spectra caused by magnetic gradients or RF pulses.

Current efforts in the development of PET-MRI scanner have focused on small animal systems, because the technical requirements for such systems are less demanding and applications are readily apparent [25]. However, one commercial vendor, Siemens, has already showcased the first human PET-MRI system at the Radiological Society of North America Meeting in 2006 and preliminary imaging studies have been reported using a prototype PET-MRI[26]. The Siemens device uses a MR-compatible lutetium-oxalate (LSO) crystal-based avalanche photodiode detector. The field of view in a combined mode with a PET head insert is estimated to be about 20cm axially and 24cm radially. This means that this system is primarily suitable for brain, and possibly other small parts, imaging. There are many potentially useful PET-MRI applications in the brain, including neurodegenerative disorders, epilepsy and tumours. Obviously, at this time, the majority of data supporting such instrumentation comes from cross-platform correlative studies. For example, a PET-MRI correlative study reported positive relations between hippocampal atrophy and ipsilateral association cortex hypometabolism in Alzheimer’s disease [27]. Digitally performed PET-MRI coregistration was also found to increase childhood CNS tumour characterisation in 90% of the cases and can be used to obtain a more specific diagnosis with regard to tumour grading [20]. There was also a report comparing the uptake of C^11^-choline PET tracer to the choline peak on proton MR spectroscopy (MRS) in the assessment of brain tumours, suggesting that they were both helpful in the differential diagnosis of cerebral lymphoma, glioma and non-tumour lesions [28].

MRS imaging displays the relative concentrations of chemical metabolites within a small volume of interest or voxel. *In vivo* proton spectroscopy is most widely available and is used to look at biochemical alterations in cancers and in characterisation of the ‘chemistry’ of target lesions. It provides biochemical information that may be complementary to the metabolic information obtained from PET, but unlike PET, does not expose patients to ionising radiation. In clinical oncology, MRS was initially developed for assessment of human brain tumours but has since been extended to evaluation of prostate and breast cancers. The roles of proton MRS in oncology include refinement of preoperative differential diagnosis data, which can be used to guide surgical biopsy procedures, and detection and monitoring of treatment response [29].

Traditionally, MRI scanning has been limited to assessment of limited body regions due to prolonged imaging time and limited availability. The new development of Total Imaging Matrix (TIM) technology allows fast, high-resolution whole-body MRI imaging without the need for patient or surface coil repositioning. The TIM used in the Siemens MAGNETOM AVANTO scanner combines 32 independent receiver channels with 76 array coil elements that can be connected simultaneously [30]. With advancing technology and increasing demand for high quality fast imaging, whole body human PET-MRI scanner might be available in clinical practice in the not too distant future.

## HYBRID IMAGING: EVOLUTION OR REVOLUTION?

It is easy to contend that the development of hybrid imaging was merely an evolution of principles of correlative imaging developed over the past 50 years. However, there is no doubt that this technology has revolutionised the way that clinicians think about imaging. The fused image has become the preferred visualisation tool of the end-user of imaging investigations, the clinician. Although clearly better than contrast agents, the superimposition of radiotracer signals on a set of CT or MRI images with which the clinician feels comfortable, has increased confidence in the veracity of the molecular information and its pathological or physiological basis.

In the case of PET-CT, besides the aesthetic advantages, it has become clear that accurately co-registered images increase diagnostic accuracy compared to independent or side-by-side reading of PET and CT data, but more substantially increase the confidence with which abnormalities are localised. The literature evaluating PET-CT is rapidly expanding but the vast majority of preliminary and recent studies have attested to incremental diagnostic value compared to PET or CT in a wide range of malignancies (31-44). Better localisation of abnormalities can have significant management implications, particularly in areas of complex anatomy [45]. Using the CT to provide an attenuation map with which to correct emission data has been another major advantage of hybrid PET-CT scanning. Previously various radioactive sources including Ge-68 and Cs-137 [46] were used to determine the loss of energy of detected annihilation photons due to tissue attenuation as they passed through the body. Since PET image reconstruction relies currently on detection of coincident detection of 2 photons, many photon pairs must pass through the entire diameter of the body, creating significant degradation in sensitivity for deep structures and restricting quantification of regional radioactivity. Acquisition of transmission scans using radioactive sources was often a time-consuming process, sometimes occupying as much as one third of the total acquisition time in order to achieve adequate statistical quality to provide an accurate map of tissue attenuation. Although innovative techniques including simultaneous transmission and emission scanning [47] reduced this impost, the ability to acquire a whole-body attenuation map in less than 1 minute using a MDCT has dramatically reduced this component of a PET scan. Despite the higher instrumentation costs, the more rapid scan acquisition protocols available on current hybrid PET-CT scanners allows significantly higher throughput, allowing economies of scale to maintain or reduce the unit cost of individual scans.

When the authors’ PET facility began operation in 1996, a PET scan extending from the base of the brain to the mid-thigh required an emission scan of close to 1 hour and a transmission scan of around 20 minutes in duration. Since most studies were processed with both attenuation correction and iterative reconstruction, the time taken to produce an image set for analysis was often an hour or more. With such time constraints, the procedure was limited to 6-8 patients per day. Today, the same axial extent of the body can be scanned in less than 30 minutes. Some scanners are capable of less than 10-minute whole-body scan acquisition times. As a consequence, we are able to perform 15 or more scans per scanner per day. This leads to more efficient use of radiotracers with rapid radioactive decay, greater amortisation of equipment and other fixed costs, such as maintenance. It also allows more productive use of technologist, nursing, secretarial and medical staff.

**Figure 4:**
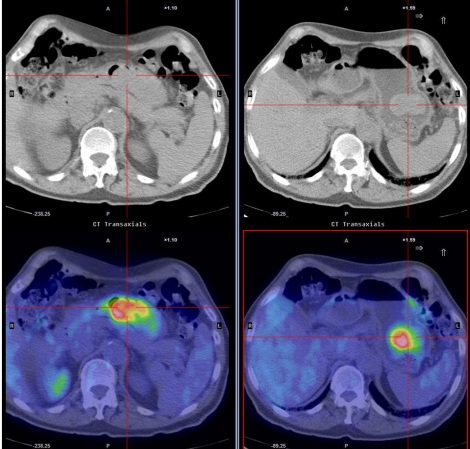
A dedicated single-bed position study acquired after whole body acquisition allows for active intervention and optimisation of CT acquisition protocols. This is particularly helpful in regions of complex anatomy like the neck and upper abdomen. These authors use this capacity for evaluation of gastric cancers, performing studies without and with gastric distension. The images on the left represent those with the stomach collapsed whereas those on the right are following gastric distension with an oral fluid load and buscopan to minimise gastric motility.

Modern computing platforms also allow almost real-time reconstruction of images. The advantages of this to patient comfort and convenience are obvious. This has been reflected in a lower likelihood of patient movement during the scan and ability to review scans while the patient is still on the bed, so that additional images can be acquired if required. This facility is routinely used in evaluating carcinoma of the stomach. A whole-body scan is first acquired with the stomach empty. This allows separation of the gastric wall from adjacent structures, including peri-gastric lymph nodes. If there is no evidence of systemic metastasis on this scan, the patient is administered buscopan as a smooth muscle relaxant and given 500 ml of water to drink in order to distend the stomach. This leads to stretching of the gastric smooth muscle, attenuating signal from it through partial volume effects, while more clearly demarking the site of the primary lesion, which being less compliant than the normal stomach, maintains its signal despite distension (Figure 4).

The greater statistical quality of CT transmission maps has also been advantageous in improving the quality of PET scans. However, the fact that the attenuation characteristics of X-rays and annihilation photons for varying tissues are significantly different means that correction factors are required to translate from a CT-attenuation map to an appropriate 511 keV map. This can lead to some discordance in quantitative analysis of tissue tracer activity [48, 49]. Soft tissue attenuation is less of a problem for SPECT reconstruction, since acquisition data in each projection are dominated by photons arising closest to the detector and thereby passing through the shortest distance of the body. Development of SPECT-CT systems has allowed more robust attenuation correction to be performed. This is most helpful in the abdomen, where the density of abdominal organs significantly reduces measured activity from deep abdominal structures, particularly in obese patients. However, because attenuation correction was not a routine part of standard nuclear medicine and SPECT was often seen as an adjunct to planar imaging due to the incremental acquisition times required, SPECT-CT has not resulted in increased throughput capacity. Accordingly, SPECT-CT is more expensive than standard nuclear medicine procedures because the equipment is more expensive than a conventional gamma camera, and to optimally leverage the diagnostic advantages of this technology it needs to be used primarily as a tomographic device rather than for high throughput planar scanning. The relatively low usage of the expensive CT component of the scanner could be viewed as wasteful. However, it is believed that flexible imaging protocols that maximise the benefits of hybrid SPECT-CT can be developed. These include having dedicated planar scanners for whole-body screening and to acquire spot views. These would then feed into a SPECT-CT scanner for dedicated regional imaging studies to better define diagnostic questions that were inadequately resolved by planar imaging.

In a hybrid PET-MRI system for whole body imaging, the issue of attenuation correction for PET using MRI images will need to be resolved. Segmentation and remapping algorithms will likely be required.

## DISADVANTAGES OF HYBRID TECHNOLOGY

**Figure 5:**
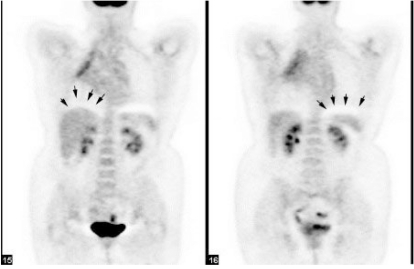
Misregistration of artefacts due to respiratory motion lead to crescent-shaped areas of relative photopenia at the level of the diaphragm. In this case of lung cancer previously treated with radiotherapy, a larger and more profound artefact is apparent in the left hemi-thorax (right panel), than on the right (left panel). presumably due to greater respiratory excursion of the untreated lung.

Although the CT and PET or SPECT components of hybrid imaging studies are acquired contemporaneously, they are not acquired simultaneously. Accordingly, this allows for movement to occur between the two scans. The most common form of movement is that associated with normal respiration [50]. It was recognised early in the development of PET that cardiac and respiratory movement significantly degraded image quality and gating was identified as a solution to this problem [51]. Because of the rapid acquisition of CT images using a MDCT it is possible to acquire images during a single breath-hold or even during normal breathing, fixing the position of structures like the diaphragm, lungs, liver and spleen that move with respiration. The emission images take minutes to acquire at each bed position and therefore respiratory blurring of the image can occur. During normal tidal volume breathing the lungs spend more of their time closer to an end-expiratory volume than to an end-inspiratory volume and certainly not at the degree of expansion associated with a forced inspiration, which is the preferred state for diagnostic CT of the chest. Thus, comparison of the CT images with the integrated PET emission image demonstrates a degree of misregistration at the level of the diaphragm although this infrequently causes major diagnostic problems [52]. The most common finding is that CT structures appear to be lower than the corresponding metabolic signal. This misregistration is also manifested in the assignment of the attenuation characteristics to structures at around the level of the diaphragm. By incorrectly assuming counts that have actually arisen within dense liver parenchyma came from aerated lung based on the CT-attenuation map, these counts are given less weighting and therefore appear as an area of relative photopenia on reconstructed images (Figure 5). Various manoeuvres have been tried to eliminate these misregistration artefacts. These have included altering breath-holding to mid-inspiration or end-expiration during acquisition of the CT component. It has been found that any instruction regarding breathing tends to significantly alter breathing pattern and therefore the authors have chosen to simply acquire the CT without any instructions to the patient other than to lie still. For situations where extremely accurate registration of anatomical and structural information is required, respiratory gating of both the CT and PET components may be an option but it places time constraints on the study and requires a more sophisticated set-up. Nevertheless, this may be worthwhile, particularly for planning treatment of basal lung cancers. Techniques have been developed to allow what is termed 4d PET-CT [53].

Gross physical movements can also occur. Making the patient aware of the need to remain still, efforts to make them as comfortable as possible and to reduce total scan time are, in the authors’ experience, the most efficacious methods of reducing patient movement. Some facilities, however, use body restraints that minimise the capacity for patient movement. Even with physical restraints, patient movements can occur and give rise to both attenuation artefacts and diagnostic difficulties related to misregistration of PET and CT data.

Unlike PET-CT, where PET and CT imaging are acquired sequentially, simultaneous acquisition of PET data and MRI images is possible in a hybrid PET-MRI scanner as the PET scanner operates within the bore of the MRI magnet. This provides for the first time simultaneous anatomical and functional imaging with not only potentially perfect fusion PET-MRI images but also the prospect of performing dynamic imaging to obtain valuable functional information.

## SHOULD HYBRID IMAGING BE AN ADJUNCT TO OR REPLACEMENT OF ANATOMICAL IMAGING?

**Figure 6:**
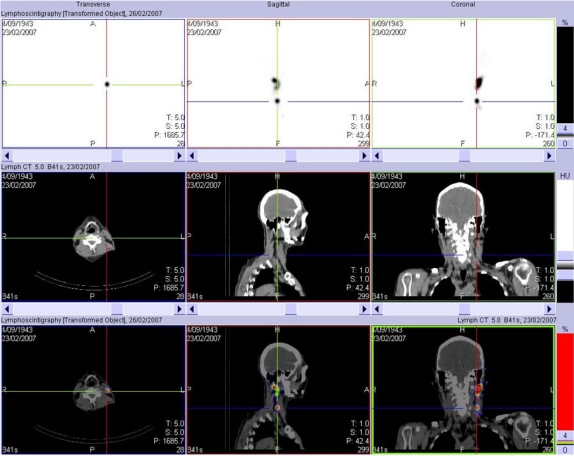
The high tumour to background ratios obtained with agents such as Tc-99m antimony colloid, used for lymphoscintigraphy, limit the anatomical localisation of abnormalities on SPECT alone. Fusion of SPECT and CT data redresses this limitation. The ability to demonstrate to the surgeon the precise location of the sentinel node means that the correct node is more likely to be sampled and that operative time and morbidity may be reduced by better surgical planning.

**Figure 7:**
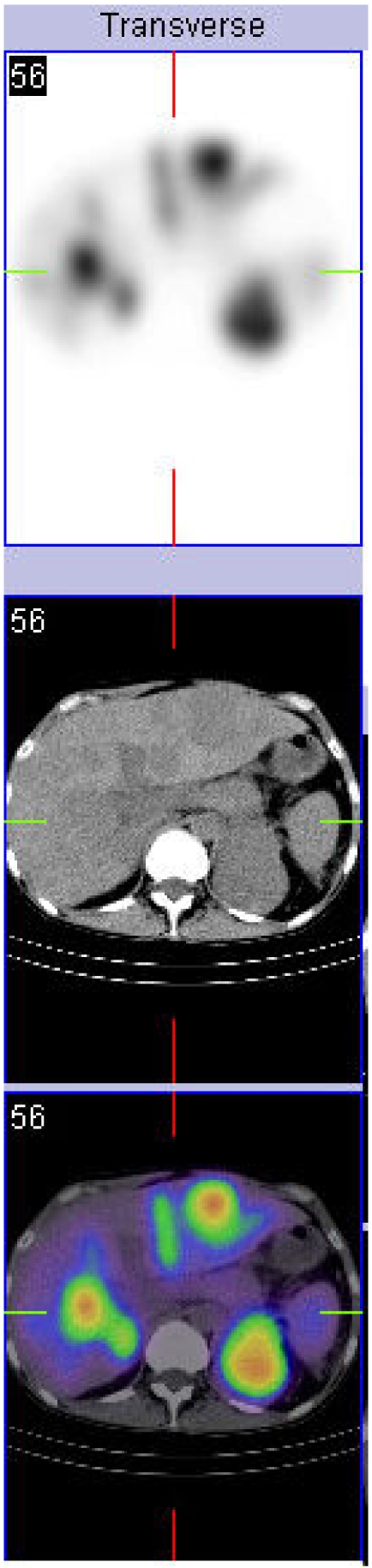
Accurate attenuation correction can aid semi-quantitative analysis of lesional uptake of therapeutic radionuclides like Lu-177 octreotate or its diagnostic pair In-11 octreotide. This can aid dosimetry planning for radionuclide therapy.

Because of the relatively high cost of PET and its restricted availability in many parts of the world, PET has been generally reserved for cases with equivocal conventional imaging results. However, in the authors’ experience, the major impact of FDG PET is to prevent futile procedures in patients in whom PET detects metastatic disease unrecognised by conventional staging techniques irrespective of whether there was any equivocation on these tests. Since most oncological therapies are selected and monitored based on the presence and extent of apparent disease, more accurate definition of these parameters is important to appropriate treatment delivery. For example, in radiotherapy patients, for whom adequate coverage of gross tumour volume is vital to achieving local control and survival, better definition of macroscopic regional nodal disease and exclusion of distant metastases would be expected to improve patient outcomes [54]. More precise characterisation of the nature and location of focal FDG accumulations identified by PET-CT is likely to further improve diagnostic performance and thereby, treatment selection and planning. By avoiding unnecessary or futile surgery, radiotherapy or chemotherapy and better assessing the response to these treatments, there is substantial opportunity to not only improve patient care but also to reduce costs, despite the higher upfront cost of the imaging component of the management paradigm if PET-CT and other hybrid imaging tests were used as the primary diagnostic test. There is increasing evidence across a broad range of indications that hybrid PET-CT [55] and hybrid SPECT-CT (56-61) are usually more accurate that either modality allow and not infrequently when compared to side-by-side comparison of each modality acquired separately. The huge number of studies being published using these technologies will continue to refine the clinical performance and role of hybrid imaging but there is no doubt that there is no turning back. The future of cancer imaging lies with hybrid imaging technologies. Because of the extremely high contrast associated with many SPECT tracers (Figure 6), the authors find that the addition of CT is even more valuable than it is to FDG PET wherein there is already substantial vicarious anatomical information by way of uptake in normal tissues. An emerging application of SPECT-CT will be the development of algorithms for radionuclide therapy dosimetry planning (Figure 7).

*Post-script note:* Although no mention of optical imaging is made above, there is preliminary work on tomographic imaging devices based on this technology [62]. The primary author is of the opinion that optical imaging will have increasing clinical application and may one day also be the subject of clinical hybrid imaging platforms.
